# Sedative and adverse effect comparison between oral midazolam and nitrous oxide inhalation in tooth extraction: a meta-analysis

**DOI:** 10.1186/s12903-023-02965-5

**Published:** 2023-05-20

**Authors:** Xia Li, Yali Liu, Chengjun Li, Jiexue Wang

**Affiliations:** Ambulatory Surgical Center, West China Shool/Hospital of Stomatology Sichuan University, Sichuan Province, No. 14 The Renmin South Road, Chengdu, 610041 China

**Keywords:** Midazolam, Nitrous oxide, Meta-analysis, Tooth extraction

## Abstract

**Objective:**

Oral midazolam and nitrous oxide inhalation were commonly used sedative and analgesic techniques during tooth extraction. It is still controversial whether oral midazolam can replace the nitrous oxide inhalation for sedative and analgesic treatment of tooth extraction. Therefore, we conducted this study in order to provide a reference for doctors to choose effective sedative and analgesic treatment in tooth extraction.

**Methods:**

We searched the Chinese and English databases including PubMed, Embase, the Cochrane Library, China National Knowledge Infrastructure, Wanfang and VIP information databases.

**Results:**

Through this meta-analysis, we found that the success rate of sedation and analgesia treatment with oral midazolam during tooth extraction was 75.67% and the incidence of adverse reactions was 21.74%. The success rate of sedation and analgesia treatment using nitrous oxide inhalation during tooth extraction was 93.6% and the incidence of adverse reactions was 3.95%.

**Conclusion:**

The use of nitrous oxide inhalation for sedation and analgesia during tooth extraction is very effective, and oral midazolam can be used as an alternative to nitrous oxide inhalation.

## Introduction

Patients will experience pain or fear during tooth extraction treatment. Studies have proved that nearly 50% of dental patients have fear disorder [1]. Especially for pediatric oral patients, due to their low ability to control their emotions and behaviors, they will show crying, struggling, anxiety and poor treatment compliance in the process of tooth extraction treatment, which will increase the difficulty of tooth extraction treatment [2]. Moreover, poor extraction treatment can cause patients to suffer from dental fear (DF). Studies have shown that 67% of adult DF patients are the result of traumatic treatment experiences in childhood [3]. Therefore, comfort in the process of tooth extraction treatment is more and more attention, sedation and analgesia technology is the main means to improve comfort in the process of tooth extraction treatment.

Nitrous oxide inhalation is a commonly sedative and analgesic technique during tooth extraction. Nitrous oxide was developed successfully in 1722. And in 1844, the American dentist Wells first used it for tooth extraction analgesia. Nitrous oxide is one of the earliest clinical application of sedative and anesthetic drugs. Nitrous oxide can stimulate neurons to release endogenous opioid peptides and activate opioid receptors and -aminobutyric acid and noradrenergic transmitter pathways to achieve analgesic effect. Through the benzodiazepine binding site to activate the beta-aminobutyric acid receptor to achieve the anxiolytic effect [4]. Nitrous oxide sedation technology can reduce pain and sensitivity and relieve anxiety by guiding patients to inhale nitrous oxide autonomously. But in the process of operation, it is easy to cause aspiration of medical staff, affecting the work and health of medical staff.

Midazolam is a commonly drug for pharmacological analgesia and sedation. Midazolam is a benzodiazepine with anti-anxiety, sedative, hypnotic, central muscle relaxation and anterograde amnesia effects. It acts as a receptor for benzodiazepine stimulation of the inhibitory transmitter GABA of the ascending reticular activating system, exerting a calming effect by enhancing the inhibition and blockade of arousal in the cortex and limbic system [5]. Midazolam is administered orally and requires patient coordination. In addition, taking midazolam orally needs to wait some time before the drug takes effect. However, at present, there are not many studies on the comparison of sedative and analgesic effects between these two drugs. It is still controversial whether oral midazolam can replace the nitrous oxide inhalation for sedative and analgesic treatment of tooth extraction. Therefore, we conducted this study in order to provide a reference for doctors to choose effective sedative and analgesic treatment in tooth extraction.

## Methods [6]

### Search strategy

We searched the Chinese and English databases including PubMed, Embase, the Cochrane Library, China National Knowledge Infrastructure, Wanfang and VIP information databases. The retrieval time was set to the database building until November 2022. The search keywords were "Dental Extraction", "Midazolam", "Nitrous Oxide". No language and the types of studies restrictions were set for this retrieval.

### The inclusive and exclusive criteria

#### Inclusive criteria

The study population was the patients undergoing dental extraction therapy. Inhalation of nitrous oxide and/or oral midazolam for sedation and analgesia were used in the studies. The success rate of sedative and analgesic treatment with nitrous oxide inhalation and/or oral midazolam and the incidence of adverse reactions were among the evaluation criteria. We have no restrictions on the types of studies.

#### Exclusive criteria

Repeated published articles. Inhalation of nitrous oxide and/or oral midazolam were not used for sedation and analgesia. The evaluation indexes of the study do not include the evaluation indexes that need to be extracted, such as the success rate, the incidence of adverse reactions, etc.

### Data extraction and paper quality evaluation

Two researchers independently extracted relevant data from the included literature, Including the first author of the article, the year of publication, the country, sample size, evaluation indicators, etc. The Newcastle–Ottawa Scale (NOS) and the Cochrane Risk of Bias Tool were used to assess the quality of the included literature. The NOS was mainly used to evaluate non-randomized controlled trials. The NOS was used to evaluate the quality of the study from three aspects: selection, comparability and exposure [7]. Cochrane risk bias assessment tool was mainly used to evaluate the quality of randomized controlled trials, from the aspects of selection bias, implementation bias, measurement bias, follow-up bias, reporting bias and other biases [8]. Finally, the quality assessment results of the literature included in this study were summarized.

### Outcome indicators

In this study, according to the common evaluation indicators in the included literature, we set the success rate of sedation and analgesia with nitrous oxide inhalation or oral midazolam during tooth extraction as the primary outcome indicator. The secondary outcome indicator was the incidence of adverse reactions of sedation and analgesia with nitrous oxide inhalation or oral midazolam during tooth extraction.

### Statistical analysis

Stata 16.0 software was used for statistical analysis of the data. According to the standard single arm meta-analysis method introduced by Jan J Barendregt et al. [9], original data included in the literature were first transformed by double arcsine method to make them conform to normal distribution and then analyzed in Stata. The double arcsine transformation formula is ${tp=sin}^{-1}\sqrt{\frac{n}{N+1}}+{sin}^{-1}\sqrt{\frac{n+1}{N+1}}$. Because converted data were used for meta-analysis, the obtained meta-analysis results needed to be restored using formula (*P* = (sin(tp/2))^2^) to get the final conclusions. In the process of meta-analysis, we used the random-effect models to analyze the data. Egger’s test was used to detect publication bias of the included literature. If *P* > 0.05, it was considered that there was no publication bias. Otherwise, there was publication bias.

## Results

### Literature inclusion

**Fig. 1:**
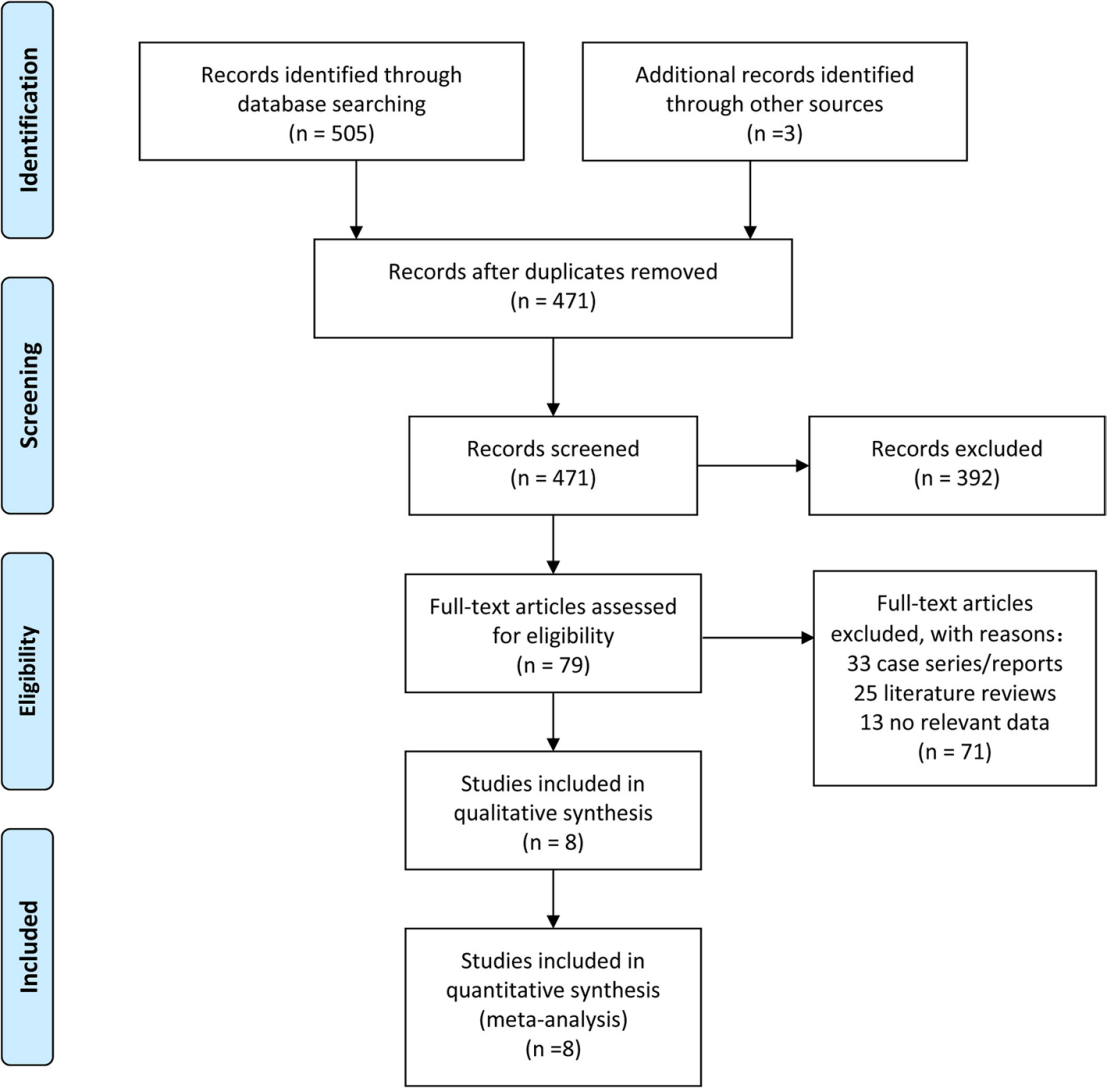
PRISMA flow diagram

A total of 508 articles were retrieved. After screening, 8 articles were finally included in this meta-analysis [10–17]. The literature screening process was shown in Fig. 1. The characteristics of the included literature were shown in Table 1.

**Table 1:** The characteristics of the included literature

First Author	Year	Country	Number of patients	Age	Therapeutic Drug	Study Designs	Literature Quality	Evaluation Indicators
K. E. Wilson [10]	2006	United Kingdom	35	5–10(years)	Midazolam Nitrous Oxide	Randomized controlled trial	HQ	Sedation and Analgesia Success Rate Adverse Effects Rate
K. E. Wilson [11]	2002	United Kingdom	44	10–16 (years)	Midazolam Nitrous Oxide	Randomized controlled trial	HQ	Sedation and Analgesia Success Rate Adverse Effects Rate
K. E. Wilson [12]	2002	United Kingdom	26	10–16 (years)	Midazolam Nitrous Oxide	Randomized controlled trial	HQ	Sedation and Analgesia Success Rate Adverse Effects Rate
Tian Xiaohua [13]	2015	China	67	4–14 (years)	Midazolam Nitrous Oxide	The cohort study	HQ	Sedation and Analgesia Success Rate Adverse Effects Rate
Shi Xiangjun [14]	2005	China	17	2–30 (years)	Midazolam	The cohort study	HQ	Sedation and Analgesia Success Rate Adverse Effects Rate
Ma Lin [15]	2012	China	46	26–84 (months)	Midazolam	The cohort study	HQ	Sedation and Analgesia Success Rate Adverse Effects Rate
Nie Juan [16]	2021	China	45	3–12 (years)	Midazolam	Randomized controlled trial	HQ	Sedation and Analgesia Success Rate Adverse Effects Rate
Liu Jing [17]	2020	China	57	6–8 (years)	Nitrous Oxide	Randomized controlled trial	HQ	Sedation and Analgesia Success Rate Adverse Effects Rate

*HQ* High Quality

### Meta-analysis results

**Fig. 2:**
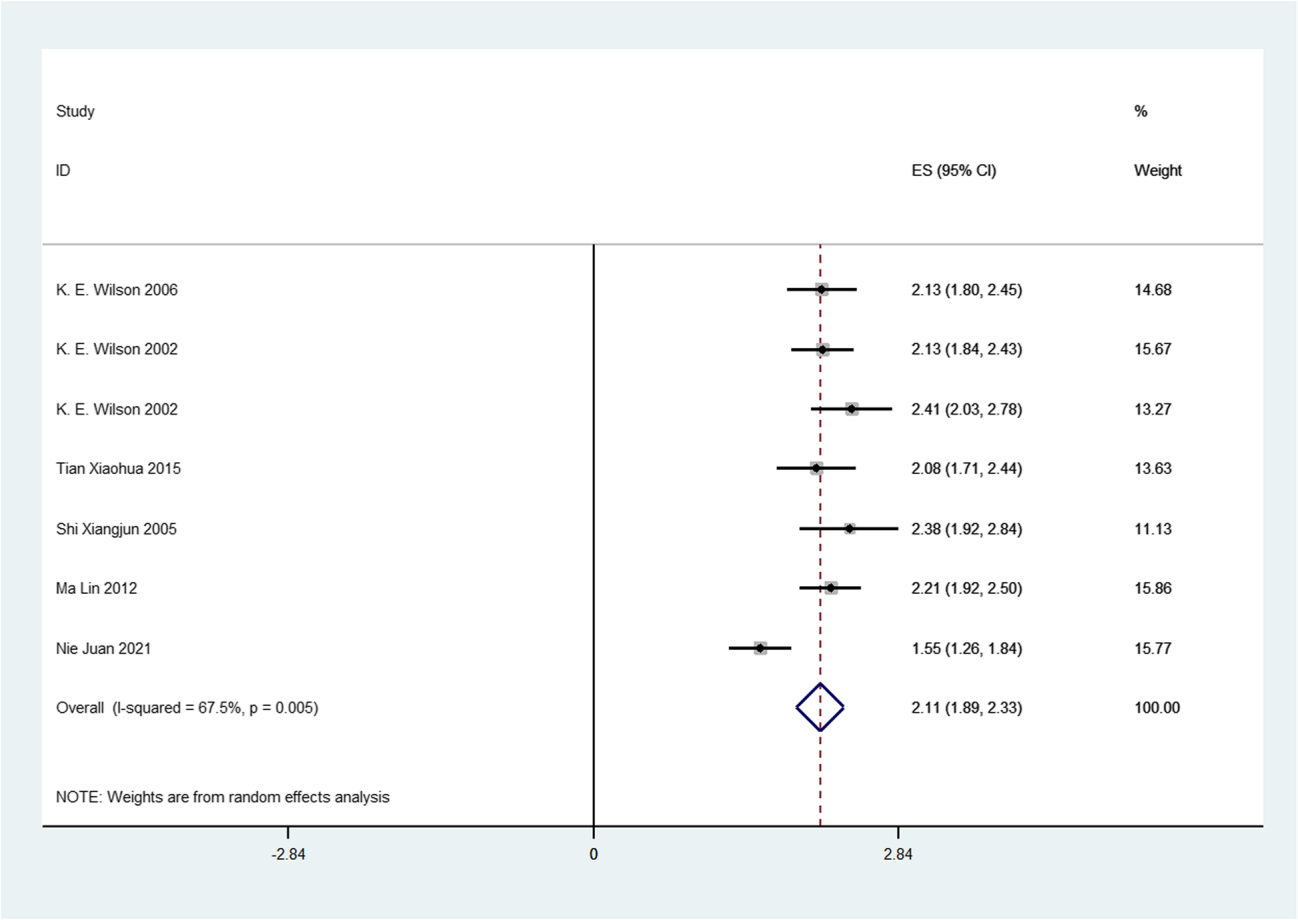
Meta-analysis of the success rate of sedation and analgesia treatment with oral midazolam during tooth extraction

**Fig. 3:**
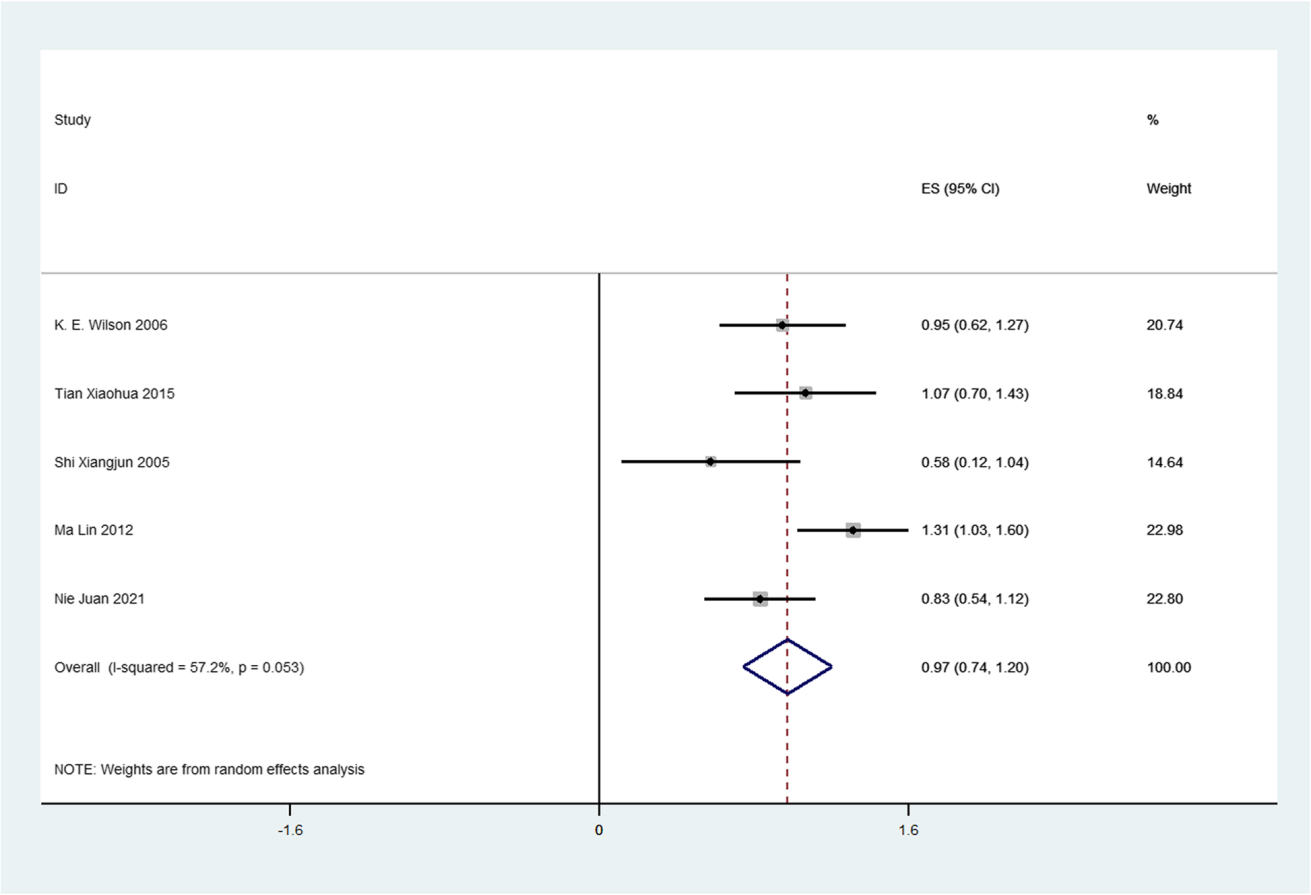
Meta-analysis of the incidence of adverse reactions of oral midazolam for sedation and analgesia during tooth extraction

**Fig. 4:**
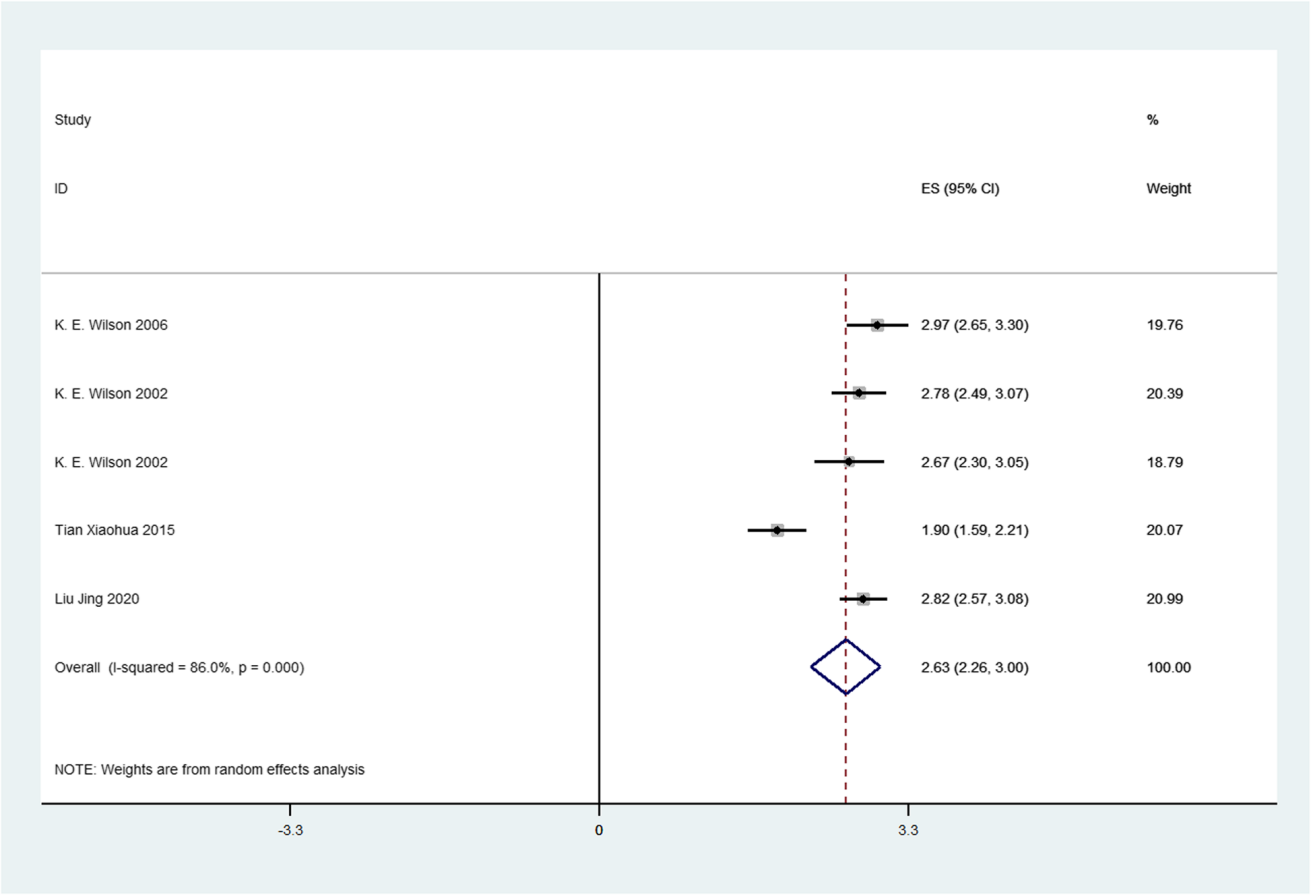
Meta-analysis of the success rate of sedation and analgesia treatment using nitrous oxide inhalation during tooth extraction

**Fig. 5:**
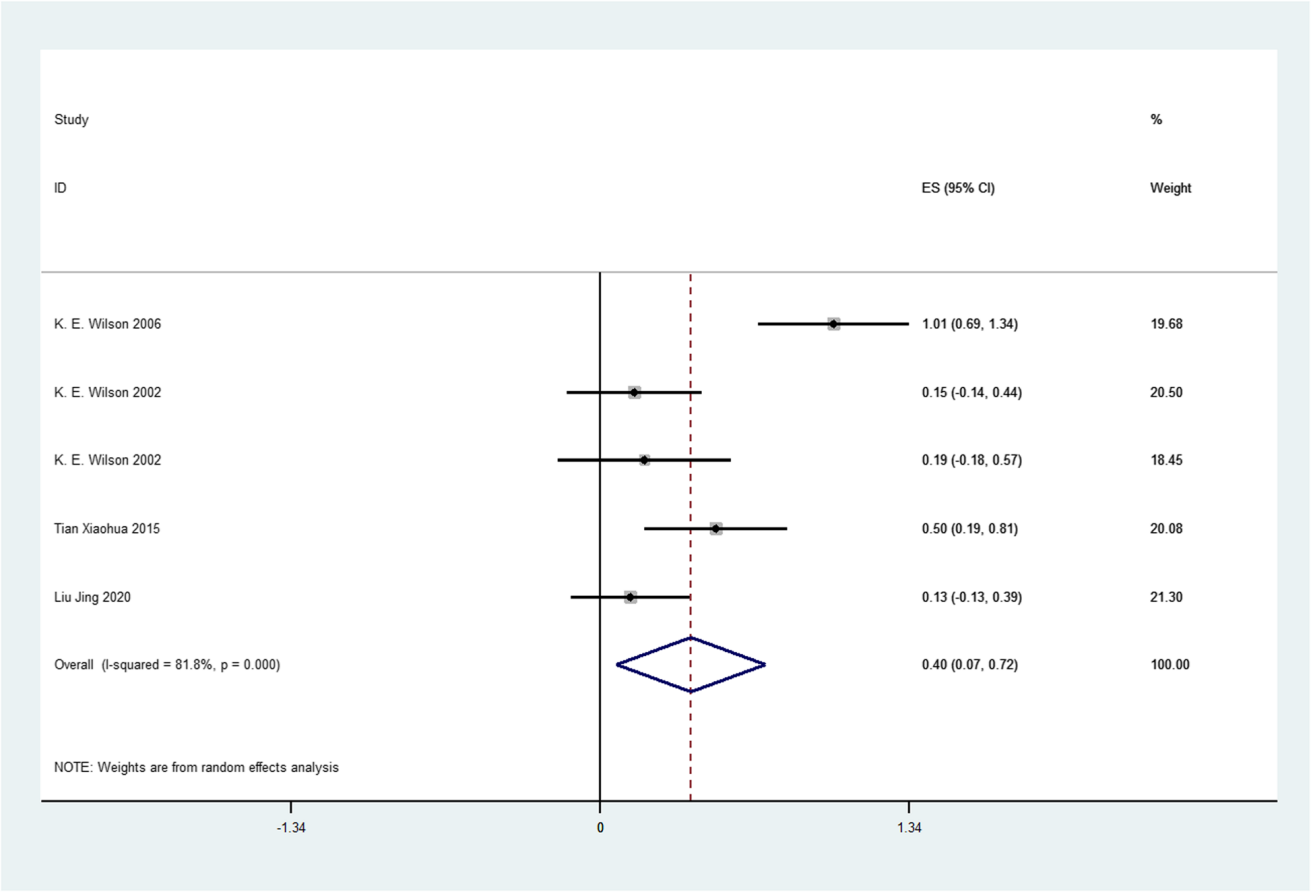
Meta-analysis of the incidence of adverse reactions in sedation and analgesia treatment with nitrous oxide inhalation during tooth extraction

**Fig. 6:**
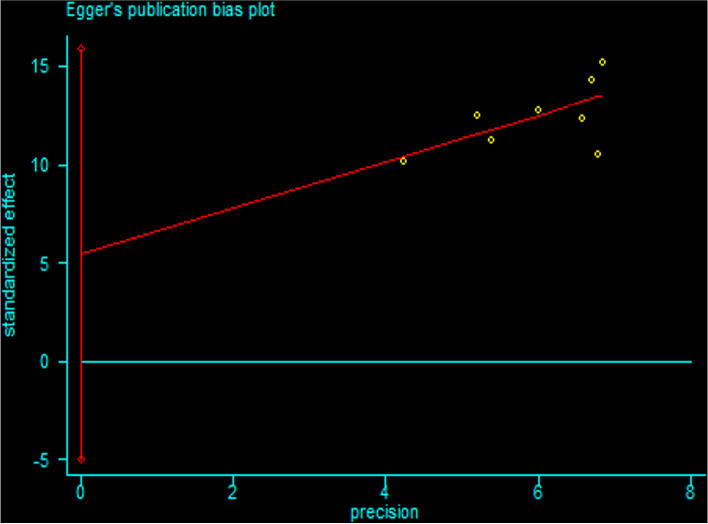
Egger’s publication bias plot

Firstly, we analyzed the success rate of sedation and analgesia treatment with oral midazolam during tooth extraction. We performed meta-analysis using the data transformed by double arcsine method, and the result was 2.11(1.89,2.33) (Fig. 2). Then the formula (*P* = (sin(tp/2))^2^) was used to restore this result, and the final result was 0.7567(0.6569,0.8442). Therefore, the success rate of sedation and analgesia treatment with oral midazolam during tooth extraction was 75.67%. Secondly, we analyzed the incidence of adverse reactions of oral midazolam for sedation and analgesia during tooth extraction. We performed meta-analysis using the data transformed by double arcsine method, and the result was 0.97(0.74,1.20) (Fig. 3). Then the formula (*P* = (sin(tp/2))^2^) was used to restore this result, and the final result was 0.2174(0.1308,0.3188). Therefore, the incidence of adverse reactions of oral midazolam for sedation and analgesia during tooth extraction was 21.74%. Thirdly, we analyzed the success rate of sedation and analgesia treatment using nitrous oxide inhalation during tooth extraction. We performed meta-analysis using the data transformed by double arcsine method, and the result was 2.63(2.26,3.00) (Fig. 4). Then the formula (*P* = (sin(tp/2))^2^) was used to restore this result, and the final result was 0.9360(0.8180,0.9950). Therefore, the success rate of sedation and analgesia treatment using nitrous oxide inhalation during tooth extraction was 93.6%. Finally, we analyzed the incidence of adverse reactions in sedation and analgesia treatment with nitrous oxide inhalation during tooth extraction. We performed meta-analysis using the data transformed by double arcsine method, and the result was 0.40(0.07,0.72) (Fig. 5). Then the formula (*P* = (sin(tp/2))2) was used to restore this result, and the final result was 0.0395(0.0012,0.1241). Therefore, the incidence of adverse reactions in sedation and analgesia treatment with nitrous oxide inhalation during tooth extraction was 3.95%. Detailed meta-analysis results are shown in Table 2. According to egger’s test, no publication bias was found (*P* > 0.05) (Fig. 6).

**Table 2:** Meta-analysis results

Therapeutic Drug	Evaluation Indicators	Results of Meta-analysis	Adjusted Results	Exact Values
Midazolam	Sedation and Analgesia Success Rate	2.11(1.89,2.33)	0.7567(0.6569,0.8442)	75.67%
	Adverse Effects Rate	0.97(0.74,1.20)	0.2174(0.1308,0.3188)	21.74%
Nitrous Oxide	Sedation and Analgesia Success Rate	2.63(2.26,3.00)	0.9360(0.8180,0.9950)	93.6%
	Adverse Effects Rate	0.40(0.07,0.72)	0.0395(0.0012,0.1241)	3.95%

The results of meta-analysis was restored using formula (*P* = (sin(tp/2))2) to reach the adjusted results

## Discussion

In this study, we used a single arm meta-analysis method to analyze the therapeutic effect of oral midazolam or nitrous oxide inhalation for sedation and analgesia during tooth extraction. Through meta-analysis, we found that the success rate of sedative and analgesic treatment with oral midazolam during tooth extraction was 75.67%, and the incidence rate of adverse effects was 21.74%. In the process of tooth extraction, the success rate of sedation and analgesia treatment using nitrous oxide inhalation was 93.6%, and the incidence of adverse reactions was 3.95%. From these data, we can know that the success rate of sedation and analgesia treatment using nitrous oxide inhalation during tooth extraction is higher than that of oral midazolam, and the incidence of adverse reactions of nitrous oxide inhalation is much lower than that of oral midazolam.

Nitrous oxide as an auxiliary anxiety control is widely used in European and American countries, and 90% of pediatric dentists have used nitrous oxide inhalation for sedation and analgesia treatment in clinical work [18]. Studies have shown that when the concentration of nitrous oxide is less than 50%, it can produce anti-anxiety and mild analgesic effects, and patients can maintain normal respiratory and cardiovascular function and normal protective reflexes [19]. In this study, we found that the concentration of nitrous oxide commonly used by dentists is 30%-50%. The time to reach the maximum sedation level after nitrous oxide inhalation is 5–6 min. The success rate of sedative and analgesic treatment with nitrous oxide inhalation was 93.6%, which showed perfect sedative and analgesia in pediatric patients. The incidence of adverse reactions of nitrous oxide inhalation was 3.95%. The main adverse reactions included drowsiness and headache, but the symptoms were mild, self-limited and do not require special treatment. However, nitrous oxide is potentially dangerous to medical staff. Chronic exposure of health care workers to nitrous oxide can cause blood, reproductive and neurological problems. Therefore, to find an alternative sedative and analgesic treatment for nitrous oxide inhalation is a hot research topic for dentists.

Oral midazolam is considered as an alternative to nitrous oxide inhalation. Oral midazolam is administered at doses of 0.50–0.75 mg / kg, and a single dose of 15 mg is safe and effective. Midazolam has little effect on respiration and circulation, and can improve the tolerance threshold of adverse stimuli in children [20]. This study found that the success rate of sedation and analgesia treatment with oral midazolam during tooth extraction was 75.67%, and the incidence of adverse reactions was 21.74%. The main adverse effects of midazolam include abnormal excitation, oversedation, and mild dose-related respiratory depression. However, the sedative and analgesic effect of midazolam alone is not ideal for children with extreme fear. The time to reach the maximum sedation level after oral midazolam was 15–30 min, and the onset rate of sedation and analgesia was lower than that of nitrous oxide inhalation. According to the data, the sedative and analgesic effect of oral midazolam during tooth extraction is acceptable and can be used as an alternative to nitrous oxide inhalation.

A total of 8 studies were included in this meta-analysis, which systematically evaluated the sedative and analgesic effects of oral midazolam or nitrous oxide inhalation in 337 patients during tooth extraction. To date, this is the first meta-analysis comparing the efficacy of oral midazolam and nitrous oxide inhalation for sedation and analgesia during tooth extraction. This study has obtained reliable conclusions through scientific and rigorous meta-analysis. However, this meta-analysis also has some limitations: The method of the single arm meta-analysis was used in this study, the data were analyzed with high heterogeneity because of the lacking of control groups. Among the 8 studies included in this meta-analysis, 5 were from China and 3 were from the United Kingdom. Chinese patients accounted for 68.84%, and whether the research conclusions are applicable to patients in other countries still needs further verification.

In conclusion, the use of nitrous oxide inhalation for sedation and analgesia during tooth extraction is very effective, and oral midazolam can be used as an alternative to nitrous oxide inhalation.

## Data Availability

The datasets generated during and/or analysed during the current study are available from the corresponding author on reasonable request.
